# Supplementary material for: Efficacy of a Protein Vaccine and a Conjugate Vaccine Against Co-Colonization with Vaccine-Type and Non-Vaccine Type Pneumococci in Mice
Source: Pathogens. 2020 Apr 10;9(4):278. doi: 10.3390/pathogens9040278 (PMC7238145; doi:10.3390/pathogens9040278)
Supplement: Supplementary file 1 [file pathogens-09-00278-s001.pdf]

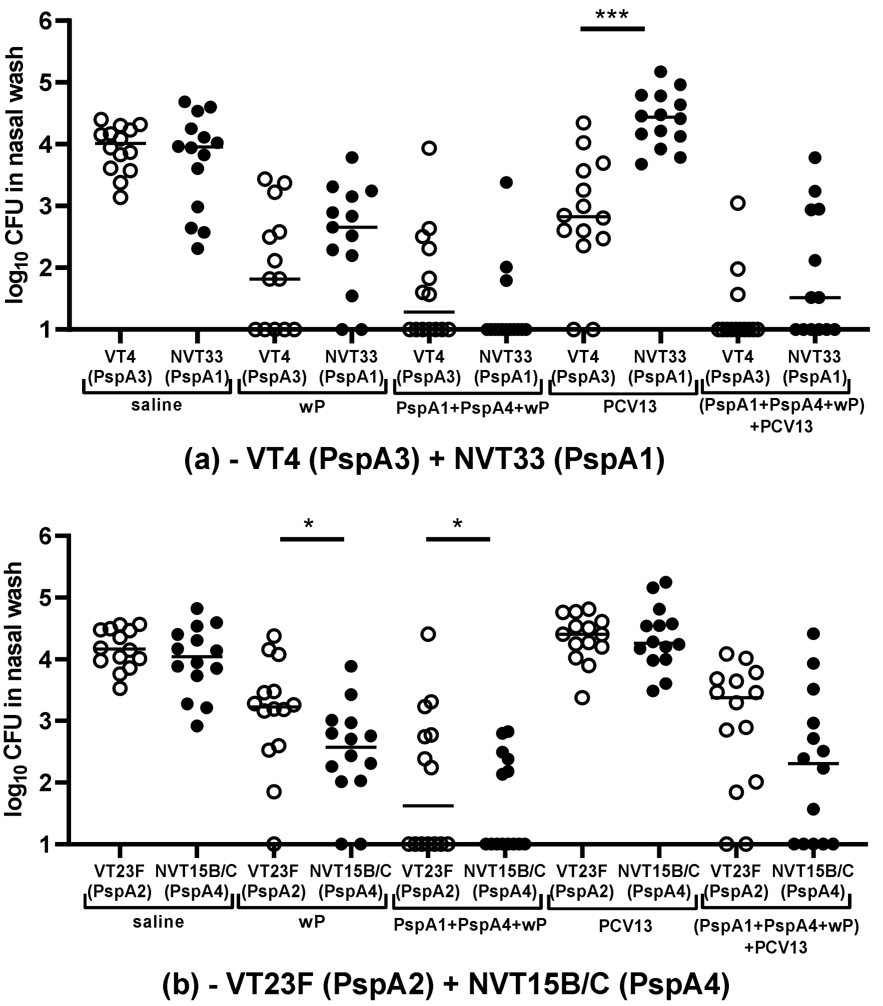

Figure S1. Paired analysis of co-colonization challenge. Mice were immunized with 3 doses of the indicated formulations and challenged with the mix of strain **(a)** VT4(PspA3) + NVT33(PspA1) or **(b)** VT23F(PspA2) + NVT15B/C (PspA4). Recovery of strain **(a)** VT4 (PspA3) and NVT33 (PspA1) or **(b)** VT23F(PspA2) and NVT15B/C (PspA4) in nasal washes performed 5 days after challenge is shown. * indicates statistical difference (Wilcoxon matched-pairs signed rank test- * $p \leq 0.05$; ***$p \leq 0.001$). Results from 2 independent experiments.
